# Climate and weather factors affecting winter sheltering by shoreline Copper Rockfish *Sebastes caurinus* in Howe Sound, British Columbia

**DOI:** 10.1038/s41598-020-71284-4

**Published:** 2020-08-31

**Authors:** J. B. Marliave, L. A. Borden

**Affiliations:** Ocean Wise Research Institute, Ocean Wise Conservation Association, PO Box 3232, Vancouver, BC V6B 3X8 Canada

**Keywords:** Marine biology, Marine biology

## Abstract

We monitored winter sheltering behavior of Copper Rockfish (*Sebastes caurinus*) in layered boulders at a shoreline in British Columbia and identified possible links to climate change and evolutionary adaptation. During late autumn and winter, these fish were inside the interstices of the boulder pile (termed “winter sheltering”); these fish were actively swimming above the boulders during spring through early fall. Sheltering duration did not vary between normal and most El Niño years (154–177 days). Sheltering longer than 6 months occurred during strong La Niña winters (197–241 days). Additionally, the proximate stimulus for entry into sheltering was intense Arctic outflow windstorms. Emergence from sheltering appears linked to water temperatures, occasionally related more to spring river flooding (snowmelt). The winter sheltering behavior we describe may be unique to shoreline populations in inland seas. Sheltering may confer a fitness advantage by conserving energy or reducing mortality from predation, thus increasing longevity and chances for successful reproduction. Our observations suggest that an ONI threshold of 0.8 °C or greater would be better suited than the current 0.5 °C threshold used to define ONI events.

## Introduction

Behavior can be an adaptive trait in terms of response to climate trends. Rockfishes are long-lived, have infrequent year-class success, and adults of inshore species tend to remain resident at home sites^1–3^, making their life history amenable to protection of broodstock by means of area closures. In 2007 164 Rockfish Conservation Areas (RCAs) were established along the British Columbia coast in an effort to protect depleted populations which reached historic lows in the 1990s (www.pac.dfo-mpo.gc.ca/recfish). One critical question associated with this RCA strategy, however, concerns the details of their residency in conscribed areas like RCAs. In particular, the literature seems contradictory and speculative regarding winter distribution of some of the inshore species^4^. In Howe Sound, however, Copper (*Sebastes caurinus*) and Quillback (*Sebastes maliger*) Rockfish are common species known to have strong fidelity to sites with high relief rock piles^5^.

Earlier research in Howe Sound found that, during winter, Copper Rockfish increase their use of interstices created by multilayered boulder piles, where they become relatively inactive^6^. This winter sheltering behavior was also documented in Puget Sound^7^. Thus, reduced densities of Copper Rockfish during fall and winter SCUBA surveys in the San Juan Islands which have been attributed to a potential depth migration^8^ might alternatively be explained by winter sheltering^4^.

In the present study, we monitored seasonal variation in Copper Rockfish use of interstices within multilayered boulders at a shallow shoreline site of Howe Sound. We used diver surveys of in-versus-out with respect to sheltering in boulders to determine the seasonal duration of sheltering over a period of 13 years. With this long-term observational, field-based data set we examined the link between local weather patterns, large scale climate indices and rockfish sheltering behaviour to demonstrate how biological data can better inform our understanding of climate patterns. A brief tagging study and records of recruitment demography elsewhere in Howe Sound are used together with survey observations to assess size demography and residency of copper rockfish at this shallow shoreline site.

## Results

**Figure 1:**
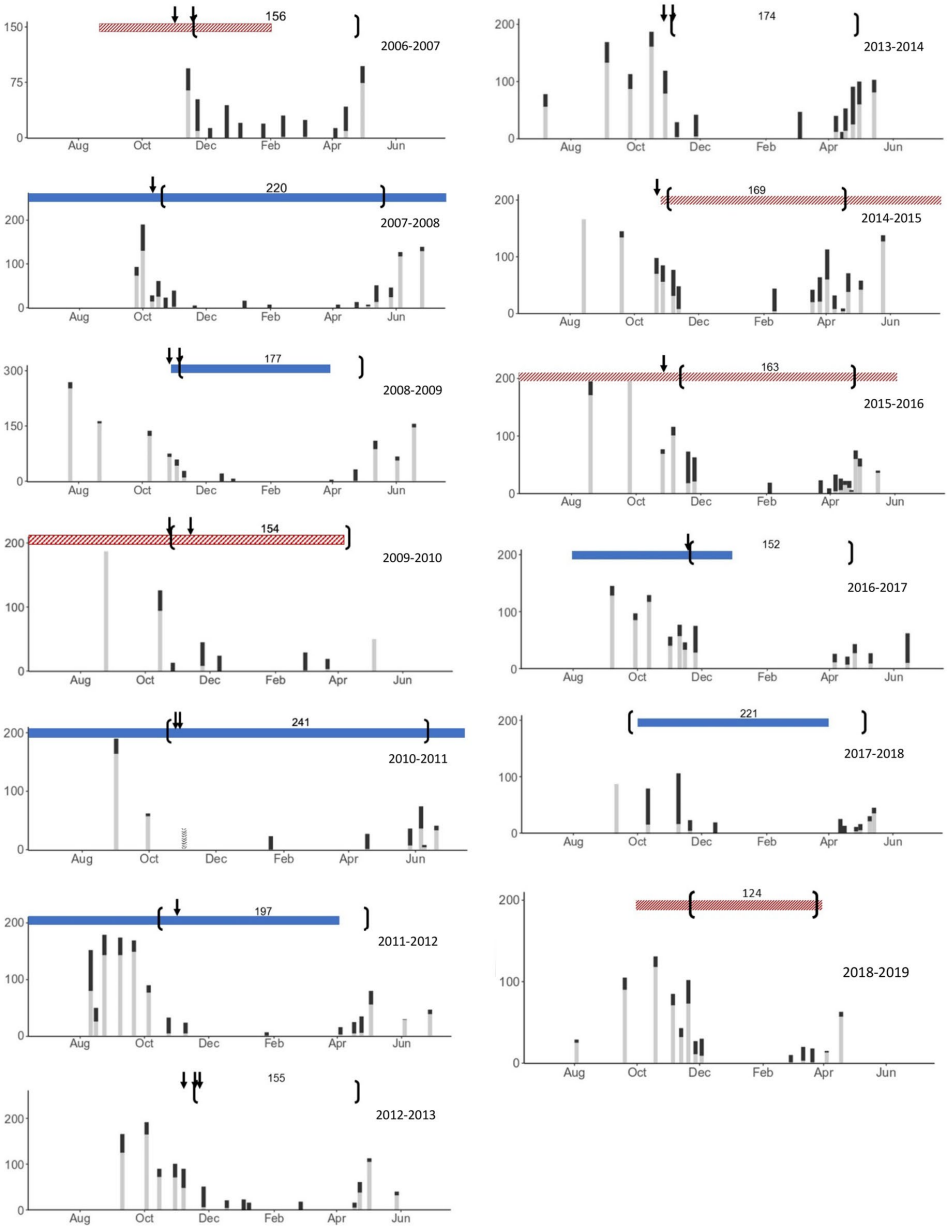
Copper Rockfish abundance at NW Bowyer Island, Howe Sound, British Columbia. Dark bars = rockfish IN; Light bars = rockfish OUT. Brackets indicate entry and exit dates from sheltering as well as the total number of days spent hiding. Arrows = windstorms. Red hatched bars = El Niño and blue bars = La Niña. Striped bar in 2010–2011 represents a lack of count data available but a note of confirmed hiding of rockfish observed by divers.

In 10 out of 13 years (exceptions 2011, 2017, 2018), winter sheltering in boulders (defined as > 50% inside rock interstices) occurred closely after strong windstorms during the autumn (Table 1, Fig. 1). Early sheltering (before Nov. 1) occurred during four strong La Niña winters in 2007, 2010, 2011 and 2017. These early entry years resulted in hiding durations of 197–241 days during winters with anomalies from − 1.6 to − 0.86 °C. Late exit from sheltering (after mid-May), occurred during the La Niña springs of 2008 and 2011. Exit was particularly late in 2011 (strong La Niña) when river flood timing was the latest in the 13 years of study (Table 1). The two weak La Niña winters (both − 0.66 °C anomalies) yielded hiding periods of 152 and 177 days, similar to normal and El Niño years, which had a range of 124–174 days. Note that the normal years had anomalies of − 0.3 °C and − 0.22 °C, close in value to the two weak La Niña events. The El Niño winters had anomalies ranging from 0.74 to 2.44 °C. Large outliers for both El Niño and La Niña ONI events prohibited statistical analysis due to non-normal distribution.

**Table 1:** Summary of Copper Rockfish winter sheltering at NW Bowyer Island, Howe Sound, British Columbia in relation to wind storms, Fraser River peak floods and the Ocean Niño Index (ERSSTv5). Anomaly calculated as average of the strongest 5 months of the ONI event from Jul-Jun encompassing the winter, and in normal years, the average anomaly for Nov–Mar.

Year	Date IN	Date OUT	Days hiding	Wind event (kph, direction)	Fraser river freshet (m^3^/s)	ONI 5mo. average anomaly
2006/07	Nov 18	Apr 22	156	Nov 1–4 (65 N) Nov 19–20 (65 S)	Jun 10 (12,100)	0.74
2007/08	Oct 13	May 20	220	Oct 7–9 (74 N)	May 26 (11,100)	− 1.5
2008/09	Nov 6	May 2	177	Oct 30–31 (50 N) Nov 5–7 (57 N)	Jun 9 (8910)	− 0.66
2009/10	Nov 10	Apr 13	154	Nov 3–5 (63 N) Nov 9 (82 S)	Jun 28 (7620)	1.34
2010/11	Oct 16	Jun 14	241	Oct 27–28 (57 N)	Jul 4 (11,600)	− 1.6
2011/12	Oct 14	Apr 28	197	None prior	Jun 23 (12,800)	− 0.98
2012/13	Nov 16	Apr 20	155	Nov 8–12 (56 N)	May 17 (10,900)	− 0.22
2013/14	Nov 4	Apr 27	174	Nov 6–7 (61 N)	May 29 (10,831)	− 0.3
2014/15	Nov 2	Apr 20	169	Oct 15/16 (55 N) Oct 21/22 (68 S) Oct 24/25 (68 N)	Jun 3 (8913)	0.84
2015/16	Nov 12	Apr 23	163	Oct 24/25 (66 N) Nov 10 (54 S)	na	2.44
2016/17	Nov 20	Apr 21	152	Nov 10/11 (62 N) Nov 14/15 (61 N) Nov 18/19 (67 N)	na	− 0.66
2017/18	Sep 26	May 5	221	None prior	Jun 17 (14,400)	− 0.86
2018/19	Nov 24	Mar 28	124	None prior	June 4 (8420)	0.82

For early sheltering in 2011 (Oct 14) there were no strong windstorms before Nov 8 (Table 1, Fig. 1). In contrast, during the El Niño winter of 2015 a strong Oct 24 windstorm did not drive fish into sheltering. Instead, a subsequent single wind event Nov. 10, 2015 appears to have driven fish into sheltering by Nov. 12.

**Figure 2:**
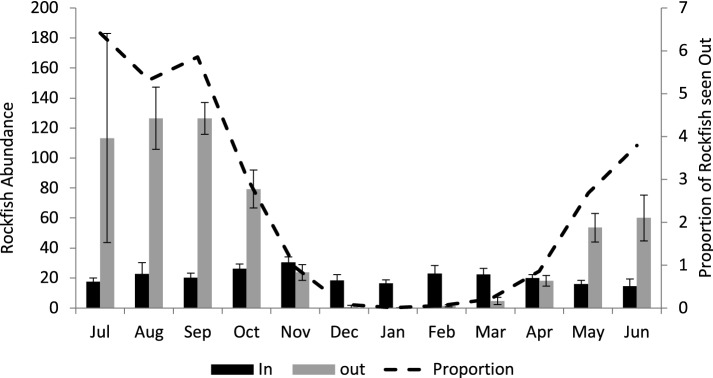
Copper Rockfish monthly abundance at NW Bowyer Island, Howe Sound, British Columbia. Dashed line indicates the proportion of rockfish ‘out’ during each month. Dark bars are rockfish seen ‘in’; light bars are rockfish seen ‘out’ (mean ± 1 SE).

The greatest reduction in overall numbers sighted was during January (Fig. 2); fish were sheltering in boulders from Nov to Apr, on average, but very few fish were “out” during Dec–Mar. The data presented in Fig. 2 shows that the total population size is about 200. Note that the numbers seen sheltering during central winter months of Dec–Feb do not show significant increases in counts compared to early or late winter because during December and January fish usually were beneath several layers of boulders. Divers were able to comment anecdotally about shallow versus deep sheltering according to how many layers of boulders they were sighting through; the deepest sheltering was mid-winter. Although very few opportunities occurred where a dive light could penetrate a direct line through several layers of boulders, fish were seen sheltering in pairs on some of those occasions. This rock pile is multi-layered enough that most fish in “deep sheltering” were out of sight lines available to divers outside the rocks, and thus could not be scored. During summer, rockfish scored as sheltering were typically just inside the outer layer of boulders and were easy to sight.

Only 6 of 102 tagged fish were sighted, but all (red and yellow tags) were within the original tagging sites, with no more than 10–15 m lateral movement evident. Loose tags were seen several times on the seabed, so shedding occurred. The first fish tagged in Nov. 2006 was sighted in Aug. 2007 before any other fish had been tagged. The only other tag sighting that spanned winter sheltering was of a tag inside the rockpile in Jan. 2010. Fish in the central boundary region (ca. 20 m wide, with no tagging effort) never became frightened at the sight of divers, whereas the fish in the two tagging sections started to flee and hide in rocks at the sight of a diver in 2008 and 2009, before the procedure was abandoned. Within a year of cessation of spear tagging rockfish ceased to flee rapidly from divers in the two tagging zones. Due to limited tag sightings, statistical analysis was not possible for tagging data.

Minimal growth was evident with large juveniles achieving small adult size during the first half decade of the study and no fish growing larger than about 25 cm by the end of the study period, during which time very few limited recruitment events for Copper Rockfish were observed in Howe Sound. Juveniles sighted in 2006–2011 were in the 17–19 cm total length size range (considered juvenile) whereas all fish sighted from 2012 onward were 20 cm total length or longer, considered adult. No fish at this site appeared to be over 25 cm total length by the end of the study.

Two other concurrent surveys were conducted in Howe Sound during this period: seabed taxon and Rockfish Abundance Survey. The taxon survey yielded 47.3% ± 14.2% SE adults (67 dives, 267 adult Copper Rockfish) for 2004–2011, versus 71.4% ± 7.7% SE adults (81 dives, 64 adults) for 2012–2019. The Rockfish Abundance Survey yielded 78.8% ± 7.7% SE adults for 2004–2011 (42 dives, 663 adults) versus 94.7% ± 1.3% SE adults (206 dives, 4,939 adults) for 2012–2019. Across both surveys there was a shift toward a predominantly adult population though in neither survey was there a significant increase in the proportion of adults in the population between the two time periods (taxon surveys: *p* = 0.17, t = − 1.48, df = 10.81; Rockfish Abundance Survey: *p* = 0.37, t = − 0.95, df = 7.77). Although not statistically significant, these results are in concordance with the observation of growth from juvenile to adult in fish from the population observed for winter sheltering on Bowyer Island.

**Figure 3:**
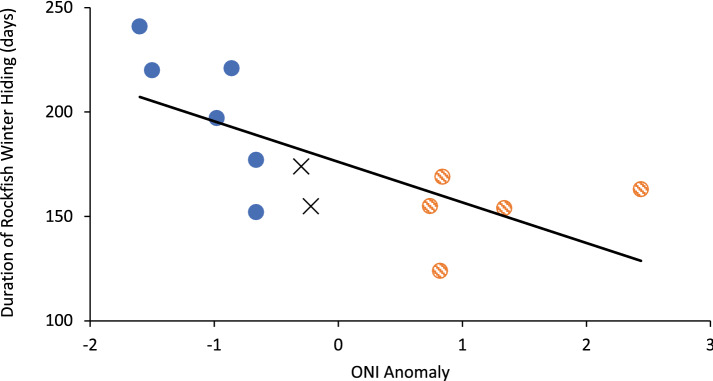
Duration of winter sheltering by Copper Rockfish at NW Bowyer Island, Howe Sound, British Columbia as a function of ONI anomaly strength. Red, hatched = El Niño event, blue = La Niña event, X = no event (normal). Line represents line of best fit (R^2^ = 0.48, *p* value = 0.009).

The anomalies listed by NOAA for ONI climate events were as low as − 1.5 for the La Niña winter of 2007/2008 and − 1.6 for 2010/2011. In those two winters sheltering lasted 220 and 241 days, respectively (normal winters − 0.3 °C and − 0.22 °C avg anomaly, 174 and 155 days sheltering). The greatest positive anomaly of the millennium was in 2015/2016, at + 2.44 anomaly (163 days sheltering). Sheltering duration during El Niños did not differ markedly from each of the two normal winters or the two weakest La Niña winters (Fig. 3). However, duration of sheltering decreased significantly with increasing ONI anomalies (R^2^ = 0.43, *p* value = 0.008; Fig. 3).

## Discussion

The present survey illustrates the use of interstices among boulders for sheltering during winter by Copper Rockfish. Copper Rockfish at this shallow shoreline reef take refuge in piled boulders for approximately 5.5 months each winter. Entry into sheltering is primarily driven by Arctic outflow windstorms, with strong La Niña events (anomalies > − 0.8 °C) linked to prolonged sheltering of over 7 months. Tagging results revealed virtually no longshore movement, and no crevice habitat exists in deeper water at this site, so we presume sheltering rather than any movement away from the piled boulders. Similarly, tagging surveys in Puget Sound showed that copper rockfish on high relief habitats have small home ranges (< 5 m^2^) compared to those found on low relief habitats^9^, suggesting that it is reasonable to consider minimal off-reef movement for the population in the present study. The greatest reduction in overall numbers sighted was during January (Fig. 2), likely related to continuing intensity of Arctic outflow winds during November through January. As winter progressed, fish were observed to move deeper into crevices where for the most part we could not detect them. Most fish were in fact sheltering too deeply (Fig. 2) for sighting except during summer and early fall (July to Oct), with far fewer fish seen outside the rocks during late spring (May, June).

In a previous study in the same region (Howe Sound), a decline in trophic interaction strength during winter^6^ was likely owing to a decline in rockfish activity levels associated with use of boulder interstices, while sheltering in deeper-water rock piles was not continuous through winter. It is unlikely that temporary sheltering affords complete protection from either predation or fishing mortality for these deeper-water populations. Winter sheltering in two other rockfish species, the Yellowtail (*Sebastes flavidus*) and Dusky (*S. ciliatus*), has been observed for the period of November through April in southeast Alaska^10^. These authors reported relative inactivity when rockfish were in crevices among boulders during this period, and further reported inactive fish observed more frequently among boulders in November and December, with none encountered during 11 of 13 dives during February and March.

Overall population size remained stable (n ≥ 200) throughout the study, with most fish at small adult size. Minimal growth was evident with large juveniles achieving small adult size during the first half decade of study and no fish growing larger than about 25 cm by the end of the study period, during which time very few limited recruitment events for Copper Rockfish were observed in this region (unpublished data). Copper Rockfish in open ocean habitats reach maturity at sizes > 30 cm total length^3^ so this Bowyer Island shoreline population remained at body sizes below average for the species through the period of study. Open coast or deep rocky reef habitats may have enough prey biodiversity to support a high enough growth rate to render winter sheltering less advantageous in those habitats compared to inlet shorelines. The present study site has high densities of juvenile Shiner Perch (*Cymatogaster aggregata*) available as prey during summer and fall, but those fish move into deep water during late fall^11^, reducing prey abundance in the shallow boulder area of the sheltering study. Copper Rockfish on the outer coast of Vancouver Island grow to larger sizes at younger ages in the open ocean, compared to fish from the heads of major inlets^12^ and these populations differentiate genetically. Similarly, in Howe Sound larger Copper Rockfish occur in deeper water where they do not spend the entire winter inside boulder piles^6^.

It is unlikely that these rockfish grew rapidly and moved to deeper water, replaced by new recruits on a continuous basis, because little recruitment was evident throughout Howe Sound during the latter 7 years of this study. The entire population grew slightly during the 2006–2010 period and fish remained at relatively small adult sizes through 2019, with a stable population size. Arctic outflow windstorms can be associated with rapid temperature drops from snowmelt events, such that the rockfish sheltering in shallow interstices could experience bradycardia and cardiac functional adaptation^13^ that would induce torpor conducive to sheltering. Advantages of winter sheltering in terms of longevity may, in this type of shallow site exposed to winter storms, be greater than any fecundity advantages of more rapid growth on deeper reefs, given the rarity of strong reproductive success^3^. The tendency to shelter through the entire winter may be a selected trait that improves fitness in certain habitat types (shoreline boulder slopes or cliff crevices in coastal inlets). Certainly, residence within the boulder piles protects the fish from the surge of winter Arctic outflow winds, which are more extreme during La Niña events^14^. Sheltering would also conserve energy during the pregnancy period of the reproductive cycle. Fish coming out of sheltering in spring were occasionally observed to be pregnant. It is noteworthy, however, that on one occasion in late spring when the population was still sheltering a pregnant female was observed in a shallow interstice, upside down among boulders with her belly and genital papilla oriented skyward. For the continuous sheltering in the shoreline rockpile in this study, mortality due to predators or fishing during the winter sheltering period may be minimal. Pinniped predators are present through the period when fish are out but they evacuate the area during winter outflow windstorms, so sheltering from predation is not likely as a stimulus. The advantages of sheltering could contribute to longer life spans of rockfish exhibiting this unusual behavior. Greater longevity would increase the odds of reproducing in the relatively infrequent years favorable to larval survival.

A comparison of the patterns observed in annual entry and exit from sheltering with respect to ONI events showed that sheltering duration appears to only increase with ONI anomalies of ± 0.8 °C or more, more so for La Niña events than for El Niño. Currently, ONI anomalies of 0.5 °C are utilized for defining ONI events. It may be that a higher threshold for defining ONI events may better represent the biological patterns observed here. Longer carry-over effects of successive ONI events may also affect duration of sheltering over a period of several years. For example, brief sheltering of 152 days occurred in winter 2016/2017 during a La Niña event that was preceded by the greatest El Niño of this time series; whereas, the second of two consecutive La Niña events in 2017/2018 coincided with the long sheltering period of 241 days. Note that the brief sheltering during the first winter of observation (2006/2007) occurred during the third consecutive El Niño of 2002–2007, with only one intervening, weak La Niña event. Arbitrary use of 0.5 °C as cut-off for significant ONI events could be improved by using biological data to verify decisions about significance of these climate events.

Seabed biodiversity data have been used to support a model for identifying climate regime shifts where anomalies for both El Niño and La Niña exceed 1.0 °C on the ONI scale^15^. Biodiversity accurately reflects climate regime shifts^16^ as in the case where Bering Sea groundfish biodiversity marked a 1989 climate regime shift^17^. A study of all macroscopic seabed biodiversity for Howe Sound diving sites over a period of decades suggests that climate regime shifts involve average ONI anomalies on the order of 1.0 °C or more rather than 0.5 °C^15^. In the present study, only 2 of 13 winters (2012/2013, 2013/2014) qualify as normal using present anomaly value of 0.5 °C; however, if anomalies ≥ 0.8 °C are used then 6 of 13 winters were normal (2006/2007, 2008/2009, 2014/2015 and 2016/2017). In terms of unusual duration of sheltering of 197–241 days, only four of the six La Niña winters qualify; the two La Niña winters that would not qualify under the revised minimum average anomaly yielded relatively normal sheltering periods of 177 and 152 days. Anomalies of 0.5 °C or greater qualify as ONI events on the ONI ERSSTv5 tables, but the Copper Rockfish only seem to attend to average anomalies of − 0.86 °C (La Niña) or 0.82 °C (El Niño) or greater with respect to significant behavioral responses.

In conclusion, the winter sheltering behavior of these Copper Rockfish could be linked genetically to their small body size, as for small, old fish in west Vancouver Island inlets^12^. If so, then Howe Sound would seem to have a balanced polymorphism for body size and winter sheltering since much larger Copper Rockfish reside on deeper reefs in Howe Sound where sheltering within high relief boulders is only occasional through winter^6^. Further, the 13 years of the present study seemed not to involve any strong recruitment events to this small population, which has stayed very stable in numbers. The stable numbers suggest that the sheltering does reduce mortality risks for the duration of winter. For future assessment of MPA and RCA impacts on rockfish demography, this current study needs to be replicated with addition of more invasive methods. Animals need to be sampled for ageing and for molecular genetic analysis according to the methods^12^ used on West Coast of Vancouver Island inlets. The sheltering documented in this present study will most likely be found to occur where extreme Arctic outflow winds are most severe, in Jervis, Toba, Bute and Knight Inlets as well as in Dean Channel and Portland Canal^18^, as well as in SE Alaska^10^. Those regions could be predicted to confer advantage to rockfish sheltering deep in interstitial spaces in rockpiles and cliff crevices during windstorms. The winter sheltering indicates highly localized home range conducive to RCA protection, which fits the generalization that this species has relatively small home range (< 10 m^2^) over high relief habitat^3^.

## Methods

Results of 2004/2005 dives surveying Copper Rockfish^5^ revealed lowered counts during winter months and thus a winter sheltering survey was undertaken to test the observation^7^ of winter sheltering by Copper Rockfish. From 2006 to 2019 SCUBA surveys were conducted when ocean conditions were safe, year-round, around midday, at a shallow (0–8 m) shoreline site composed of multilayered boulders at the Bowyer Island RCA (49° 25.78 N, 123° 16.48 W). A total of 179 survey dives was conducted over the 13-year study. Consistency of survey effort was impacted by unsafe boating and diving weather (notably winter wind storms). Other priority research projects resulted in reduced survey effort, notably in 2010–2011 and during busy summer field research seasons. Efforts were made to ensure surveys were conducted approximately bi-weekly from late October through late November and early April through early May to ensure entry and exit timing was accurately captured. Surveys were conducted with two divers that swam 10 or 20 min, parallel transects of the lower and upper sections of the boulder pile while thoroughly inspecting crevices and holes with dive lights. Rockfish were scored according to whether they were outside crevices or sheltering (inside crevices or holes = not detectable in video transect parallel to shore). Entry into winter sheltering was defined as the median date between the last count where > 50% of Copper Rockfish were recorded as outside the boulder pile and the first dive where < 50% were recorded outside. The same method was used to determine exit from winter sheltering. Subsequent dives were conducted soon after transition for confirmation.

To investigate site fidelity of Copper Rockfish at the Bowyer Island RCA, rockfish were tagged with colored spaghetti tags from fall 2006 through 2009. Only one tag was inserted in Nov. 2006. In 2007, 80 fish were tagged during three non-survey dives in Sept. and Oct., then 19 fish were tagged during two dives in July of 2009. The divers would slow to a near stop and stalk individuals before spearing the tag into the dorsal musculature above the spinal column. We implanted totals of 53 red tags and 49 yellow tags in fish; we inserted all yellow tags at 10–30 m north of the marked reef mid-point, and we inserted all red tags at 10–30 m south of the mid-point. However, fish became wary of the spear sling so that significant tagging was only achieved in 2007. Due to the invasive nature of the tagging, this aspect of the study was terminated in 2009. Monitoring of rockfish movement into and out of the rockpile continued for another 10 years.

Through the period of this sheltering survey, our team continuously conducted two other surveys in Howe Sound that yielded data on Copper Rockfish demography. A seabed taxon survey^15^ monitored occurrence of all visible seabed taxa with estimates of rank abundance, including 148 dives (from 2004 to 2019) that yielded data on Copper Rockfish. We also conducted a Rockfish Abundance Survey (https://ocean.org/wp-content/uploads/Rockfish-Survey-Instructions.pdf) in which we collated data on counts of rockfish according to life stage (young-of-year, juvenile < 20 cm TL, adult ≥ 20 cm TL) for 248 dives, 2004–2019. Results of these surveys for 2004–2011 versus 2012–2019 are presented as percent adult ± SE. Fish were considered adult if ≥ 20 cm total length . Juvenile versus adult counts were based on divers referring to a marking of 20 cm on their gloved hand and ranking fish as juvenile if < 20 cm total length.

Wind speed data from Environment Canada’s Howe Sound Pam Rocks weather station (49° 29.27 N, 123° 17.97 W) (https://climate.weather.gc.ca/) was utilized to determine windstorm events (consecutive days of wind speeds reaching ≥ 50kph). Peak flood data is from Environment Canada’s Fraser River at Mission location (https://wateroffice.ec.gc.ca/), using max discharge to determine peak flood date. The Oceanic Niño Index (ONI, = El Niño Southern Oscillation, ENSO) events were classified using the ERSSTv5 data in the Niño 3.4 region (https://origin.cpc.ncep.noaa.gov/products/analysis_monitoring/ensostuff/ONI_v5.php). Anomaly values for ONI events were calculated as the average of the strongest five consecutive months of the ONI event from July to June. In contrast, normal year values were calculated based on the central five consecutive months encompassing the winter hiding period, November to March.

Duration of Copper Rockfish winter sheltering as a function of ONI event strength was analyzed using a linear regression. Proportion of adult copper rockfish in Howe Sound based on surveys from the Pacific Marine Life Surveys database from 2004–2011 versus 2012–2019 was compared using a two-sample *t*-test. An identical *t*-test was performed on copper rockfish data from the Rockfish Abundance Survey for 2007–2011 and 2012–2019. All statistical analyses were performed in R 3.5.2.

The tagging protocol was approved by the Animal Care Committee of the Vancouver Aquarium (now Ocean Wise) Board of Directors. The Board’s Research Committee approved the ethics of the entire winter sheltering project. All methods were carried out in accordance with relevant guidelines and regulations involving animal studies.

## Data Availability

The datasets generated during the current study are available from the corresponding author on reasonable request.
